# Designing Shape Morphing Behavior through Local Programming of Mechanical Metamaterials

**DOI:** 10.1002/adma.202008617

**Published:** 2021-08-02

**Authors:** Franziska Wenz, Ingo Schmidt, Alexander Leichner, Tobias Lichti, Sascha Baumann, Heiko Andrae, Christoph Eberl

**Affiliations:** ^1^ Fraunhofer Institute for Mechanics of Materials (IWM) 79108 Freiburg Germany; ^2^ Fraunhofer Institute for Industrial Mathematics (ITWM) 67663 Kaiserslautern Germany; ^3^ Fraunhofer Institute for Chemical Technology (ICT) 76327 Pfinztal Germany; ^4^ Institute for Microsystems Engineering Albert‐Ludwigs University of Freiburg 79110 Freiburg Germany

**Keywords:** homogenization, material design, mechanical metamaterials, multiscale simulation, programmable materials, shape morphing

## Abstract

Shape morphing implicates that a specific condition leads to a morphing reaction. The material thus transforms from one shape to another in a predefined manner. In this paper, not only the target shape but rather the evolution of the material's shape as a function of the applied strain is programmed. To rationalize the design process, concepts from informatics (processing functions, for example, Poisson's ratio (PR) as function of strain: ν **=** *f*(ε) and if‐then‐else conditions) will be introduced. Three types of shape morphing behavior will be presented: (1) achieving a target shape by linearly increasing the amplitude of the shape, (2) filling up a target shape in linear steps, and (3) shifting a bulge through the material to a target position. In the first case, the shape is controlled by a geometric gradient within the material. The filling kind of behavior was implemented by logical operations. Moreover, programming moving hillocks (3) requires to implement a sinusoidal function ε_*y*_ **=** sin (ε_*x*_) and an if‐then‐else statement into the unit cells combined with a global stiffness gradient. The three cases will be used to show how the combination of mechanical mechanisms as well as the related parameter distribution enable a programmable shape morphing behavior in an inverse design process.

## Graphical abstract

Different kinds of shape morphing are realized with programmable materials. Therefore, processing functions as well as if‐then‐else‐conditions are implemented in unit cells. Local geometrical adaptions allow controlling the global shape in dependency of a load amplitude. Combining these elements, (strain‐dependent) shapes as well as a moving hillock are created within a material.

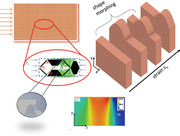

## 1 Introduction

Often the shape of a device can be optimized for one specific functionality, but many applications serve conflicting functions. For example, airplane wings being efficient during cruising and easy to lift off can only be realized by morphing concepts.^[^ ^1^, ^2^, ^3^ ^]^ The implementation of specific shape morphing behavior in devices has been studied on many scales and for different external triggers.^[^ ^4^ ^]^ On the one hand there are many approaches on the system level where morphing is often actuated by electric motors,^[^ ^3^, ^5^ ^]^ piezoactuators^[^ ^6^, ^7^ ^]^ or multi‐material systems, for example, bi‐metals.^[^ ^8^ ^]^ On the other hand, shape morphing is realizable through an adaption of the geometry of the micro‐structure. This can be done on the atomistic scale, for example, using phase changes and gradients, as well as on a *μm* ‐ *cm* level. For many years, linear effective properties such as Poisson's ratio (PR) and Young's modulus have been engineered in materials.^[^ ^9^ ^]^ Greaves et al.^[^ ^10^ ^]^ present an overview of structures and achieved properties. Elasticity tensors for extremal materials have been shown in the 1990s,^[^ ^11^ ^]^ but their practical implementation is mainly driven by the evolution of manufacturing technologies in recent years. In metamaterials, periodically arranged unit cells whose properties overcome the ones found in nature are designed^[^ ^12^, ^13^ ^]^ (e.g., negative PR^[^ ^14^, ^15^, ^16^ ^]^ and high stiffness‐to‐weight‐ratio^[^ ^17^ ^]^). Moreover, additive manufacturing allows to easily change geometric features (beam thickness, angles) of the unit cells locally in the material. This approach enables a non‐uniform distribution of material properties in so called graded materials that can result in different shapes during loading.^[^ ^18^, ^19^, ^20^, ^21^ ^]^ Designing shape morphing behavior requires not only to control constant properties but also to control the way they evolve, for example, a strain‐dependent PR.

**Figure 1:**
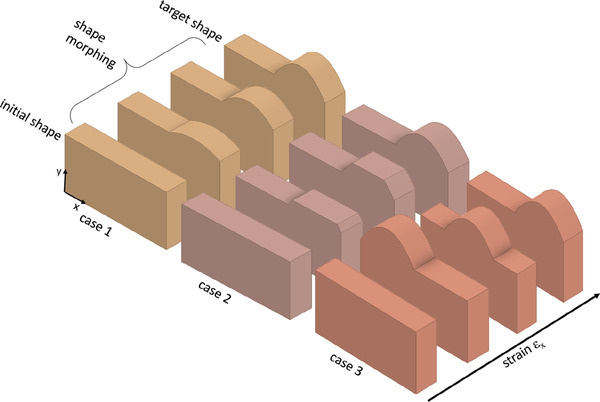
Schematic representation for three cases of shape morphing behavior under axial strain ε_*x*_. Case 1: The target shape is reached by (linearly) increasing the amplitude of the lateral deformation. Case 2: The filling of a target shape is adjusted until a complete fitting of the target is achieved. Case 3: A shape (bulge) is shifted through the material until the target position is reached.

In this article, we propose different ways of integrating mechanical mechanisms in the unit cells which lead to various non‐linear elastic (but still controlled) behavior. This behavior can be described in terms of processing functions and if‐then‐else conditions. The cells have been assembled to macroscopic materials and the functions and conditions are locally adjusted through the adaption of the geometric parameters of the unit cells. The combination of the different properties (stiffness, PR) which are distributed in the material lead to a specific shape perpendicular to an applied load, also shown in refs. [^18^, ^19^]. Moreover, the logical statements allow us a global programming of the material's shape. In the following, we show three cases how to transform from an initial to a target shape under increasing strain (see **Figure** **1**). In the first case, a target shape is obtained by linearly increasing the amplitude of the lateral deformation. In the second case, the target shape is filled up in linear steps. In the last case, the target shape is achieved by shifting the position of a bulge in the material. To realize the first case, we need a unit cell that processes a linear function depending on one tunable geometrical parameter. For the second case, an if‐then‐else condition that can be tuned by a geometrical parameter was implemented in a unit cell. The third case requires a cell that processes a sinusoidal, non‐monotonic function in combination with an if‐then‐else condition as well as global stiffness gradient in the material.

## 2 Results and Discussion

**Figure 2:**
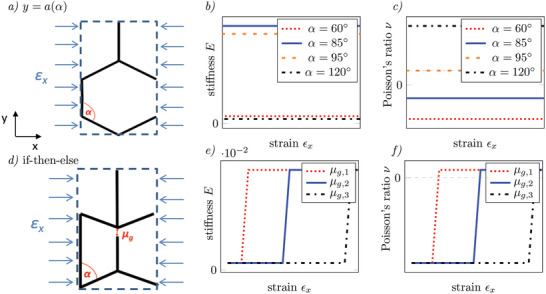
Two unit cells and their adjustable properties stiffness *E* and PR ν in the regime of small deformations. a) Geometry of the hexagonal cell. b) and c) resulting constant stiffness and PR for different angles α over strain ε_*x*_. d) Geometry of the cell with inner contact. e) and f) Resulting stiffness for different gap widths μ_*g*_ (μ_*g*, 1_ < μ_*g*, 2_ < μ_*g*, 3_) for fixed α. Under increasing strain, we see an abrupt change in the properties that can be interpreted as an if‐then‐else condition.

**Figure 3:**
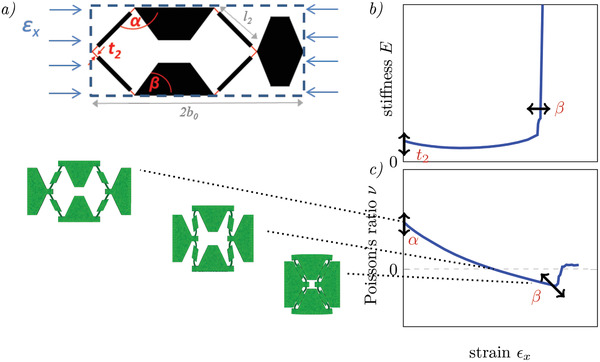
Adapted hexagonal cell with hinges showing a changing PR in the large deformation regime, three programming parameters: angle α, contact angle β, hinge thickness *t*_2_. a) Geometry of the cell; b) (strain‐dependent) stiffness showing an if‐then‐else‐behavior; c) PR that evolves from positive to negative values and deformation states of the cell (left). The arrows visualize how the curves can be shifted by changing the geometrical parameters α, β, and *t*_2_ within the unit cell.

**Figures** **2** and **3** show the three unit cells that exhibit the required behavior (functions, if‐then‐else conditions) for the three aforementioned cases. They can be parametrized and assembled into metamaterials. Their complete geometrical description can be found in Section S1, Supporting Information. To achieve reversible behavior, we consider a purely elastic base material without plastic deformations. Global shape morphing requires to orchestrate the local deformation in a way that the target shape can be achieved when the metamaterial is deformed. One approach is to design the local PR that corresponds to a shape morphing behavior shown in Figure 1, case 1. For the sections of the part, where the PR is negative, the part contracts under compression and vice versa which can be designed in the unit cells. The hexagonal structure (Figure 2a) is well‐known from literature^[^ ^22^ ^]^ and has properties that strongly depend on the angle α. In the case of small deformations (ε << 1), the mechanical properties of such structures can be calculated analytically and are constant for a chosen angle. Such a representation was derived on the basis of classical homogenization concepts^[^ ^23^ ^]^ (see Section S2.1, Supporting Information). The resulting linear stress‐strain relation leads to constant stiffness and PR for small deformations. Figure 2b,c shows the derived Young's modulus *E* and PR ν for different angles α and a constant unit cell size. The variation of this angle allows to generate a broad range of properties only dependent on one geometric parameter. For α < 90°, the cell adopts the shape of a bow‐tie and is auxetic. Choosing α > 90° leads to a honeycomb with positive PR.^[^ ^15^, ^16^, ^18^, ^19^, ^24^ ^]^ Note that the adaption of the PR is accompanied by a change in the stiffness and vice versa. These properties can be decoupled through adapting other parameters such as beam thicknesses (*t*) or the base material.

Another possibility to design a target shape into the metamaterial is locally limiting the maximum deformation under a given load (see Figure 1, case 2). This can be achieved by abruptly increasing the local stiffness at a defined unit cell deformation. To gain these non‐linear properties, we introduced a contact gap that can close and open during load cycles and that leads to a structural transformation (Figure 2d). The cell geometry changes between a bow‐tie and one consisting of two trapezoids. This transformation can be considered in the homogenized model by introducing an internal variable δ that allows to distinguish between open and closed contact (see Section S2.1, Supporting Information). The linear stress–strain relation is now divided into two parts with different slopes. The constant properties of case a) establish an if‐then‐else condition:

(1) $$\begin{array}{c} \begin{array}{l} if\;\varepsilon_{x}>\varepsilon_{\lim}(\mu_{g})\;then\;\nu ≃0,\;E=E_{2}(\alpha )\;\\ else\;\nu =\nu_{1}(\alpha ),\;E=E_{1}(\alpha )\;\;\;with\;E_{2}>>E_{1} \end{array} \end{array}$$

Figure 2e,f shows the change in PR and stiffness. The condition (strain under which the structural change takes place, ε_lim_) depends on the initial gap μ_*g*_. The constant functions (*E*, ν) in the if‐then‐else‐condition are controlled with the parameter α (here: α = 60°). While the stiffness strongly increases, the PR jumps from negative to positive values close to zero. The auxecity is suppressed by the structural change. For more complex behavior, for example, introducing a hump on the surface which can be moved along the loading axis (see Figure 1, case 3), different mechanisms must be combined. Here, complex interactions between unit cells need to be programmed in the metamaterial. To design a suitable unit cell, the properties and geometric parameters in the former examples can be used as starting conditions. The cell must be extended so that large deformations are possible (see Figure 3a). In this case, the behavior cannot be derived analytically anymore but may be approximated with semi‐analytical equations as well as numerically investigated using a finite element model (see Section S2.1, Supporting Information). We start from a honeycomb cell but change some geometric parts: We include hinges at the connection of the beams that allow them to rotate. The hinges are realized in terms of tapering of beams at the connection points. They are parameterized by the thickness *t*_2_ that is smaller than the middle part of the beam *t*_2_ < *t*. In consequence, the deformation is located in these parts of the beams. Additionally, a contact element has been implemented which is characterized by the angle β. It has a similar function as μ_*g*_ in the upper example but blocks the rotation of the beams.

The angle α changes during uniaxial compression and the PR ν becomes a non‐linear function (Equation 2) of the strain depending on the current angle (α_0_ + Δϕ) of the cell geometry (beam length *l*_2_, cell width 2*b*_0_, details can be found in Figure S1b, Supporting Information). Δϕ describes the difference between α_0_ (angle in the undeformed geometry) and α(ε_*x*_) during loading. This leads to a non‐monotonic lateral contraction visualized in Figure 3c. At low strains, the PR is positive ((α_0_ + Δϕ) > 90°) and changes to negative values for large strains ((α_0_ + Δϕ) < 90°). The deformation is then stopped through the contact that increases the stiffness and sets the PR approximately on zero. So three different ways of shape changing under load are combined in one cell: extension, zero deformation, and contraction. We can describe the cell's behavior as following:

(2) $$\begin{array}{c} \begin{array}{l} if\;(\alpha_{0}+\Delta \phi )<\beta \;then\;\nu =f(\Delta \phi ),\\ E=f(\Delta \phi ,t_{2})\;else\;\nu =≃0,\;E=E_{2} \end{array} \end{array}$$

(3) $$\begin{array}{c} \text{with}:\;\Delta \phi =(\alpha_{0}-90^{\circ })-\sin^{-1}\left[\frac{2\varepsilon_{x}b_{0}}{2l_{2}}+\sin(\alpha_{0}-90^{\circ })\right] \end{array}$$

Equation 3 is based on a semi‐analytical description assuming pure rotation of the beams which gives a rough estimation of the unit cell behavior (see Section S2.1, Supporting Information). Figure 3b,c shows the resulting stiffness and PR from the FEM simulation. The above‐described parameters α, β, and *t*_2_ can be used to control the processing functions. We can increase the stiffness by increasing *t*_2_, move the shift in the stiffness to lower (higher) strains by increasing (decreasing) β, and influence the start value of the PR through the parameter α. This allows to adapt single cells in an assembly.

**Figure 4:**
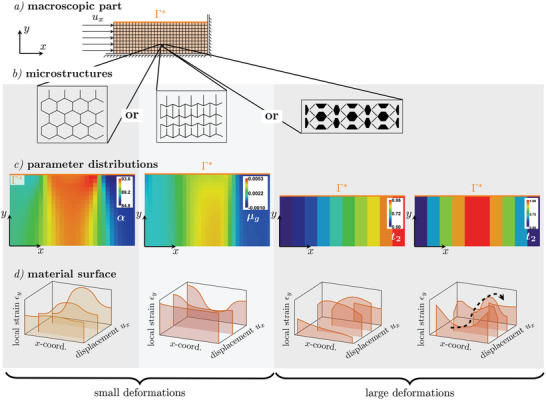
Analysis of materials consisting of the three unit cells with locally programmed parameters. a) Load case and boundary conditions used for all examples, b) schematic microstructures, c) distributions of the geometrical parameters within the unit cells over the material's cross section: (left) the angle α was varied in the honeycomb structure (unit cell Figure 2a), (middle left) the contact gap μ_*g*_ was varied in the structure with contact (unit cell Figure 2d), (middle right) the hinge thickness *t*_2_was varied linearly, and (right) sinusoidally in a structure consisting of cells shown in Figure 3. d) Surface deformations corresponding to the local strain ε_*y*_ over the *x*‐coordinate of the sample for different global displacements *u*_*x*_ resulting in linear (left), piecewise‐linear (middle left), and non‐monotonic (middle right) and sinusoidal (right) morphing behavior.

In a next step, macroscopic shape morphing structures were realized by creating 2D arrays of the unit cells shown in Figures 2 and 3 within a FEM framework. In the first structure, we varied the geometrical parameters α in a macroscopic material that consists of the hexagonal cells (Figure 2a). The corresponding data is displayed in **Figure** **4**, first column. The second structure is composed of the cells shown in Figure 2d with a fixed angle α and a variation of the contact gap μ_*g*_ (Figure 4, second column). For the third case, we show two examples that consist of the cell shown in Figure 3. The parameters α and β were fixed in both examples but *t*_2_ varied linearly and sinusoidal over the *x*‐coordinate of the samples (Figure 4, third and fourth column).

Figure 4a shows the macroscopic scale with the applied boundary and load (details in Figure S3, Supporting Information), Figure 4b the underlying structures, Figure 4c the parameter distributions in these structures, and Figure 4d the resulting surface shapes under different strain amplitudes. Here, we plotted the *y*‐strain over the *x*‐coordinate which corresponds to the shape of the sample surface Γ* (marked with an orange line).

The parametrization allows us to control the behavior (linear, if‐then‐else, non‐monotonic function), described in the unit cell section, locally and thus to program the global shape morphing. If we aim to achieve a specific shape morphing behavior in a material, we need to know how to adapt the geometric parameters in the individual cells by taking into account their respective stress/strain field. Due to the homogenized model of the hexagonal cell with and without contact, the ideal parameter distribution can be determined with optimization methods (for small deformations). To use this continuum descriptions for the materials, the size of the unit cell must be much smaller than that of the macroscopic specimen so that the macroscopic strain does not vary significantly over the length of a cell. However, comparison of the results for the fully resolved structure and the homogenized model reveals that, for the case in Figure 4a, good results can be achieved with as few as nine cells over the height of the structure, despite the fact that the angle varies strongly among the cells (see Figure S4, Supporting Information).

To gain a target shape, we aim to optimize, in particular minimize, the error or difference to a certain desired surface displacement (in the lateral direction) under compression. The error is also referred to as cost or objective function and can be specified as follows:

(4) $$\begin{array}{c} J(u)=\left\|u-\bar{u}\right\| \end{array}$$

where *u* and $\bar{u}$ are the actual and desired displacements, respectively. Here, *u* and $\bar{u}$ are functions of space, hence J(u) measures the error in the Euclidean norm. In order to minimize this error, we define γ as control, which is the design parameter as function of space. Therefore, we may write *u* = *u*(γ). For instance, we may consider the angle α, the contact gap μ_*g*_, or the hinge thickness *t*_2_ in Figure 3 as control through the design. Note that in this work, we focus on only one control variable, more precisely we optimize w.r.t. to one design variable (e.g., either α or μ_*g*_, see Figures S4 and S5, Supporting Information). Nevertheless, optimizing with one design variable is still non‐trivial, due to large number of unit cells in compound and, hence, large design space: Since γ is also a function of space, each cell (or finite element) is equipped with a degree of freedom within the parameter space. Therefore, optimizing γ within a compound domain means the simultaneous optimization of the design parameter in each unit cell. An adjoint sensitivity approach is a convenient choice for solving such optimization task. Since we want to apply an iterative gradient descent method,^[^ ^25^ ^]^ the functional or Fréchet derivative $J^{\prime }(u(\gamma ))\delta \gamma$ is necessary. Roughly spoken, it may be understood as directional derivative with *δγ* as variational or virtual term. In order to shorten the notation, we use the abbreviation

(5) $$\begin{array}{c} d_{\gamma }J(u(\gamma )):=J^{\prime }(u(\gamma ))\delta \gamma \end{array}$$

In this work, we apply the adjoint sensitivity approach; that is, before each gradient descent iteration, the original mechanical balance of equilibrium and its adjoint version have to be solved. For these two steps, we use the finite element software *CalculiX*.^[^ ^26^ ^]^ The resulting solutions are then processed for the evaluation of the total parameter derivative $d_{\gamma }J$. Roughly, one can describe an iteration like this:

(6) $$\begin{array}{c} \gamma^{i}=\gamma^{i-1}-d_{\gamma }J(u(\gamma^{i-1}))\;i=1,2,3,\cdots \end{array}$$

The iteration above is repeated until convergence occurs (Details in Section S3, Supporting Information).

In our first example, the honeycomb cell was used and the parameter α was varied in the range of 60° to 120° to have positive PRs in some regions as well as negative in others. Hence, the lateral contraction is non‐uniform over the *x‐coordinate* of the sample but its amplitude is linear in the applied horizontal global displacement *u*_*x*_ (Figure 4d, left). As we optimized the parameters, we could reduce the range to values between α = 84° – 94° (Figure 4c, left). Ref. [^18^] as well as [^19^] report 3D‐printing and analysis of similar structures (but for large deformations) resulting in similar shapes as shown in Figure 4d, left. However, we choose a multiscale approach which allows us to treat the structures as materials (with a lot of unit cells) and to transfer the methodology to more complex problems (e.g., include more parameters). This case shows how a predefined target shape, with an amplitude depending on the load, can be achieved through an adaption of geometrical parameters.

The second structure (Figure 4b, middle) is built out of bow‐tie cells (α = 60°) with an inner contact (Figure 2d). The contact gap μ_*g*_ was locally adapted in the following. The achieved lateral strain (ε_*y*_) over the *x*‐coordinate of the part looks similar to the first example, but results in a non‐uniform (in this case negative) lateral contraction. If we try to relate the lateral contraction to the load amplitude, we do not get a linear relation anymore. Figure 4d, left, shows different shapes for different applied global displacements *u*_*x*_. This is a result of the local if‐then‐else conditions implemented in the unit cells. The local stiffness change leads to roughly one order of magnitude stiffer regions. They deform less under increasing amplitude of the global displacement and in addition have a positive PR. From then on, these cells are (locally) stress‐controlled and their shape remains practically unchanged (but the others cells can still be deformed). In consequence, the surface displacement is a piecewise‐linear (non‐smooth but continuous) function of the global displacement. Every time a unit cell changes its contact state, the global shape changes as the smooth deformation is blocked. When all contacts are closed, the overall stiffness of the material increases significantly.

For this metamaterial, it is possible to optimize both the surface displacement and the overall stiffness but also to control a deformation path. In Figure 4d, middle left, we show how the shape changes from flat to non‐smooth, to a “u‐shape.” These structures are interesting for applications in which stiffness as well as shape should be adaptable (friction change, packaging, etc.). The optimization of μ_*g*_ leads to a narrow parameter distribution in the material (Figure 4c, left), wherein the unit cells must be adapted only slightly. Intended stiffness changes can be found in few metamaterial structures. Fang et al. show an overall stiffness change in a self‐locking origami structure^[^ ^27^ ^]^ and in ref. ^[^ ^28^ ^]^ externally controlled electromagnets have been used to open and close structures. Another possibility is to exploit instabilities to have a structural transition.^[^ ^29^, ^30^ ^]^ Moreover, bistable structures can also lead to such changes combined with memory behavior as shown in ref. [^31^] and ref. [32]. These approaches lead to noncontinuous functions due to the snap‐through. Also in granular system, we can introduce stiffness changes due to particle jamming.^[^ ^33^, ^34^ ^]^ However, our structure reacts passively under strain control, is simple to realize, and allows continuous deformations as well as a local implementation. We only design a jump in the local properties (ν, *E*) but not in the displacements. So we avoid instable systems which are complicated to handle, but could be useful to change abruptly between two different shapes. In this example, the shape is not only programmed by the choice of the initial geometry but also by the loading amplitude.

**Figure 5:**
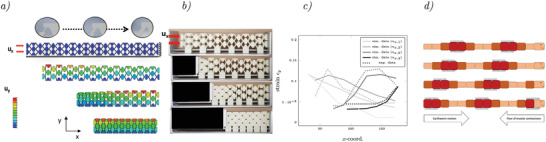
Realization of a structure with nonlinear behavior: a) FEM simulation of the structure, displacement *u*_*x*_ increasing from top to bottom. b) Corresponding deformations of our additive manufactured demonstrator. c) Comparison of the experimental and simulation data. d) Peristaltic of an earthworm redrawn from ref. [5].

In the third case, the aim is to program a mechanical metamaterial to form a surface hillock similar to the first example, but now, this hillock should move along the *x*‐axis depending on the applied strain in that direction. This requires to implement a non‐monotonic behavior and to consider large deformations. For this reason, we used the unit cell shown in Figure 3 that can expand as well as contract and assembled an array of 2×10 cells. The hinge thickness was defined in each cell so that the stiffness increases from left to right (*t*_2_ = *t*_2,0_ + *t*_2,0_ *x*, Figure 4c, middle right). The cells are connected in series (with increasing local stiffnesses) and in parallel (with equal local stiffnesses). The non‐monotonic, strain‐dependent stiffness of the unit cell leads to a global stiffness minimum which moves through the material. The deformation of the structure starts on the left and migrates to the right when the stiffness of the left part increases. The non‐uniform local strain leads to different lateral contractions in the cells depending on *t*_2_. Due to the local non‐monotonic behavior of the single cell, a non‐linear, non‐monotonic behavior of the system can be achieved. Figure 4d, middle right, shows the different surface morphologies, which can be controlled with this structure. If we choose the hinge thickness according to a sinusoidal distribution (*t*_2_ = *t*_2,0_ + *t*_2,0_sin (*xπ*), Figure 4c, right), the surface can even have two bulges that merge together after a certain strain (Figure 4d, right). The variation ranges from wedged and warped to a plane surface shape although the design space is not widely explored yet. Videos of the FEM simulations of the two structures are provided as Videos S7 and S8, Supporting Information. In the future, our homogenization and optimization framework shall be expanded to include large deformations, history dependency, and more geometric parameters to handle this complex behavior. Just like in the second example, the initial geometry as well as the amplitude of the global displacement can be used in this structures to program the shape. Moreover, the more complex unit cell geometry allows a larger range of target shapes possible within one sample. Producing such kind of materials is challenging due to their complex, inhomogeneous geometries and the difference in size between device volume and unit cell ($\frac{unit\;cell\;volume}{device\;volume}<<1$). A first approach was made by 3D‐printing structures of a few unit cells, but there are still a lot of limitations like nozzle sizes and overall build volume. Nevertheless, we produced a demonstrator part made of the thermoplastic elastomer (TPE) *Elastollan C78 A15* corresponding to the structure shown in Figure 4, third column, with a linear distribution of the parameter *t*_2_ (see Section S4, Supporting Information). As an experiment, the demonstrator has been fixed on a plate so that a rolling contact in *x*‐ and *y*‐direction occurs. This permits a qualitative validation of the simulation results. In **Figure** **5**a,b, finite element simulations (with *ABAQUS 2018*,^[^ ^35^ ^]^ Section S2.2, Supporting Information) are shown next to the 3D‐printed part under different load conditions. Figure 5c shows the comparison of the shapes of the printed samples and FEM simulations. Here, we used the local lateral strain over the *x*‐coordinate of the sample under different global displacements (*u*_*x*,1_ < *u*_*x*,2_ ...) to describe the deformation and shape of the sample (see Video S7, Supporting Information). The experimental data was obtained by optical evaluation. To gain a better quantitative accordance complexer material models, the tolerances in the 3D‐printing process as well as the non‐ideal boundary conditions in the experimental evaluation have to be considered.

## 3 Conclusion

All in all, mechanical metamaterials open up a multiscale design space which can only be utilized if the functionality can be related to their inner structure and vice versa. We have shown that programming elements can be used in the design process of materials to obtain predefined behavior. Designing programmable behavior into such metamaterials requires to introduce logical operations (e.g., if‐then‐else), as well as the capability of processing functions (e.g., ε_*y*_ = *f*(ε_*x*_)), which can be parametrized. The selection of such elements as well as the optimization of parameter distributions is required to integrate certain (shape morphing) functions into materials. Besides controlling the final shape of a deformed material, the course and intermediate shapes can be programmed as shown in cases 2 and 3. With increasing number of controllable parameters within the unit cells, complex, non‐linear system behavior can be implemented. This level of control allowed us to create a material that behaves similar to the locomotion of earthworms (see Figure 5d) which is known for its robust and space‐saving way to move.^[^ ^36^ ^]^ A locally controlled shape morphing and a material that can expand as well as contract is required. The different parts in the material have to expand and contract alternately. In the field of soft‐robotics, this peristaltic is controlled externally through, for example, magnets^[^ ^37^ ^]^ or motors^[^ ^5^, ^36^ ^]^ and results in complex technical solutions. With our approach and the material shown in Figure 5, we can transfer this functionality directly in the material and control it through the input load. We also see potential in using such materials to control transport processes on surfaces or in tubes where purely mechanical solutions would also lead to a simplification of the systems. In our example, we varied only one of the several parameters in the unit cell; however, we see potential of the exploration of multiple parameters. This could ‐ open doors to control shapes in aerodynamic applications within the material as well as designing adapted fixing systems such as plugs and pins that could also profit from local shape control. Nevertheless, we have to integrate large deformations in our framework and to deal with strain‐dependent target functions. Therefore, we need to develop a data‐based approach wherein all representative load cases will be pre‐calculated. Furthermore, the design process needs to be optimized to implement logical elements in unit cells while considering manufacturing restrictions and avoiding instabilities. Nevertheless, such instabilities can also be used as design elements.^[^ ^29^, ^38^ ^]^ Overall, our multiscale approach to use parametrized unit cells, homogenization techniques, and optimization leads to a reduction of the complexity and helps to identify programmable materials to replace complicated technical systems.

## Conflict of Interest

The authors declare no conflict of interest.

## Supporting information

Supporting Information

Supplemental Movie 1

Supplemental Movie 2

## Data Availability

The data that support the findings of this study are available from the corresponding author upon reasonable request.
