# Supplementary material for: Designing Shape Morphing Behavior through Local Programming of Mechanical Metamaterials
Source: Adv Mater. 2021 Aug 2;33(37):2008617. doi: 10.1002/adma.202008617 (PMC11469262; doi:10.1002/adma.202008617)
Supplement: Supplementary file 1 — Supporting Information [file ADMA-33-2008617-s002.pdf]

© 2021 Wiley-VCH GmbH

ADVANCED MATERIALS

Supporting Information

for *Adv. Mater.,* DOI: 10.1002/adma.202008617

Designing Shape Morphing Behavior through Local Programming of Mechanical Metamaterials

Franziska Wenz, Ingo Schmidt, Alexander Leichner, Tobias Lichti, Sascha Baumann, Heiko Andrae, and Christoph Eberl*

Supporting Information for *Designing Shape Morphing Behavior through Local Programming of Mechanical Metamaterials*

May 17, 2021

*Franziska Wenz   Ingo Schmidt   Alexander Leichner   Tobias Lichti   Sascha Baumann   Heiko Andrae   Chris Eberl**

S1. Geometry of the Unit Cells

Figure S1 shows the unit cells introduced in the main section and their geometrical parameters. The values (value ranges) can be found in Table 1. Figure S1a shows the hexagonal cell with contact element that is the base geometry for the first and second example presented in the main part. Example (1) (Figure 2a, main section) is the special case with $l_3 = 0$ such that there is no contact. Example (2) (Figure 2d, main section) is a cell with an inner contact but α fixed to 60°. Figure S1b shows the geometry of the third example (Figure 3, main section).

S2. Modeling and Simulation

S2.1 Unit Cells

The first and second unit cell can be described with beams and a homogenized model can be attained as follows: In a finite element framework, using structural beam elements with two displacement and one rotational degree of freedom, the total stiffness matrix K of the structure is readily available. The periodicity requirement allows eliminating the degrees of freedom of half of the boundary nodes in favor of the components of the 'macroscopic' strain tensor ϵ. Collecting these in a vector $\vec{\epsilon} = [\epsilon_x, \epsilon_y, \epsilon_{xy}]$ and denoting the vector of the remaining degrees of freedom by $\underline{U}_r$, the elastic energy of the structure may be written as

$$W = \frac{1}{2}(\underline{U}^T K \underline{U}) = \frac{1}{2}\underline{U}_r^T K_r \underline{U}_r + \underline{U}_r^T K^* \vec{\epsilon} + \frac{1}{2}\vec{\epsilon}^T K^{**} \vec{\epsilon} \tag{1}$$

where matrices K_r, K^*, K^{**} result from rearranging terms of the original stiffness matrix K. Vector $\underline{U}_r$ may be expressed through the macroscopic strain by requiring the corresponding nodal forces to vanish.

$$\frac{\partial W}{\partial \underline{U}_r} = K_r \underline{U}_r + K^* \vec{\epsilon} = \underline{0} \rightarrow \underline{U}_r = B\vec{\epsilon} \tag{2}$$

which leads to

$$W = \frac{1}{2}\vec{\epsilon}^T \underbrace{(K^{**} - K^{*T} K_r^{-1} K^*)}_{V\mathbb{C}} \vec{\epsilon}. \tag{3}$$

The term in the brackets represents the effective elastic stiffness matrix $\mathbb{C}$ (multiplied by the cell's volume V) of a material with the considered structure. It is thus available analytically in terms of the geometry parameters listed in Table 1.
History dependence of the effective behavior may by introduced by considering the gap between the innermost beam's right end point and the vertex next to it. The gap can be open or closed in the course of deformation, depending on the history of the strain ϵ. When it is closed, it is assumed that the beam is linked by a flexible joint to the vertex, in which case it can only transmit compressive forces. The stiffness matrix for the closed gap case may be obtained by the procedure described above, considering now

symbol	parameter	recommended values
$2h_0$	cell height	const.
$2b_0$	cell width	const.
l_1	beam length	$b_0 + l_2 cos(\alpha)$
l_2	beam length	$h_0/sin(\alpha)$
t	beam thickness	const
t_2	hinge thickness	$< t$
α	angle between l_1 and l_2	$40° - 130°, > \beta$
β_0	angle of contact element	$0° - 90°$
μ_g	contact gap	$< 2b_0$
$\Delta\phi$	angle change for hinge stiffness	$< \alpha - \beta$

Table 1: Parameters of the unit cells.

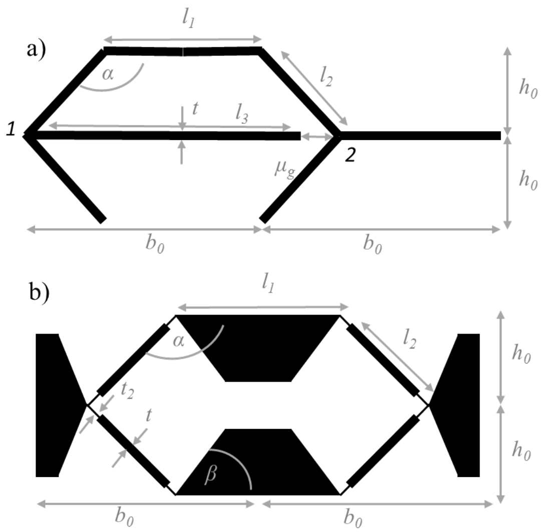

Figure S1: a) Parametrized hexagonal cell with a contact element (beam l_3) and b) cell that allows non-monotone behavior under large deformations. Parameters see table 1 (left).

the corresponding contributions of beam l_3. The constitutive behavior of such a 'material with internal contact' can be described by introducing an internal variable δ which is the difference between the distance between nodes 1 and 2 and the undeformed length of beam l_3 (Figure S1a). Open (closed) contact is then identified by δ >0 (δ <0) , and in the undeformed state $\delta(0) = \mu_g$. The macroscopic stresses are then defined by

$$\vec{\sigma} = \mathbb{C}_1 \vec{\epsilon}, \;\; \delta > 0 \tag{4}$$

$$\dot{\vec{\sigma}} = \mathbb{C}_2 \dot{\vec{\epsilon}}, \;\; \delta < 0 \tag{5}$$

and, observing that the nodal displacements depend on the strain according to equation 2, the evolution of the internal variable δ can be expressed as

$$\dot{\delta} = \begin{cases} -\vec{b_1} \cdot \dot{\vec{\epsilon}}, & \delta > 0 \\ -\vec{b_2} \cdot \dot{\vec{\epsilon}}, & \delta < 0 \end{cases} \tag{6}$$

which completes the constitutive description. As with the stiffness matrices, vectors $\vec{b_1}$ and $\vec{b_2}$ are available analytically in terms of the geometry parameters of the unit cell. The formulation is implemented in an Abaqus UMAT routine to analyze the unit cells' behavior presented in Figure 2 of the main text. In both examples the base material's Young's Modulus was set to $E = 1000$ MPa. For the hexagonal cell $\alpha = 60°,\ 85°,\ 95°$ and $120°$ were chosen. The inital contact gap in the second example was defined relative to the distance of node 1 and 2. Here the factors $c_g = 5 \cdot 10^{-4}$, $1.5 \cdot 10^{-3}$ and $3 \cdot 10^{-3}$ were used and μ_g calculated as follows:

$$\mu_g = c_g(l_1 - 2l_2\cos(\alpha)) \tag{7}$$

In the modified cell (Figure S1b) the non-linear behavior of the lateral contraction can be described with a semi-analytical model. We made the assumptions, that strains (ϵ_x, ϵ_y) are only caused by a change $(\Delta\phi)$ of α and that stiff beams can only rotate about a hinge with the torsional stiffness $c \sim t_2^3$. This leads to a lateral strain (parameters according to table 1):

$$\epsilon_y = \frac{-\cos[(\alpha - 90°)l_2] + \cos[(\alpha - 90°)l_2 - \Delta\phi]}{2h_0} \tag{8}$$

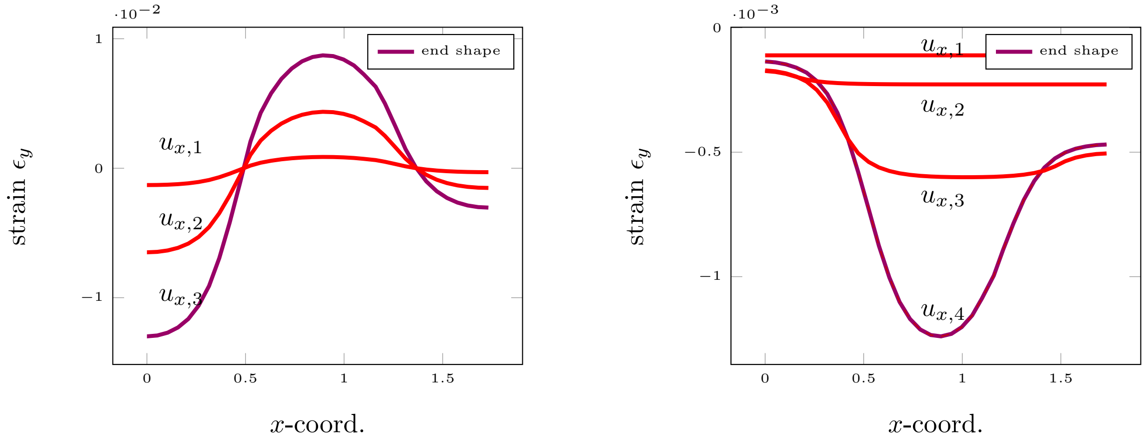

Figure S2: Lateral strain under increasing applied horizontal displacement for materials showing linear (left) and piecewise linear (right) behavior. Final shape marked in dark-red.

with

$$\Delta\phi = (\alpha - 90°) - \sin^{-1}\left[\frac{2\epsilon_x b_0}{2l_2} + \sin(\alpha - 90°)\right] \tag{9}$$

If cells are connected in series the deformation of the single cell can be calculated via a force (F_x):

$$F_x = -c\frac{\Delta\phi\ l_2}{\cos[\alpha - 90° - \Delta\phi]} \tag{10}$$

The parameter c in this equation allows to have cells with different stiffness and subsequently to operate an individual stress-strain-curve for each cell. The contact as well as the geometry of the non-ideal hinges were not taken into account here. The detailed behavior was analyzed with a FEM-simulation using the software *ABAQUS 2018* (Figure 3, main text). Linear-elastic properties of the base material have been used ($E = 1000$ MPa, $\nu = 0$).

S2.1 Materials

For the simulation of the material behavior for unit cell (1) and (2), the homogenized model described in the previous section have been used. For the third example a finite element simulation was performed due to its complexity. We used the software *ABAQUS 2018*. Linear-elastic base material properties ($E =$ 20 MPa, $\nu = 0.48$) that resemble a flexible polymer were used. The simulations were carried out with the non-linear geometry option for large deformations. The structures have been meshed with C3D8-hexahedral elements with adaptive mesh control. Contact has been modeled as "surface contact". Hard contact in normal direction and frictionless tangential behavior have been assumed. Structures of several cells have been created via a script which specifies the number of cells in x- and y-direction and the local geometrical parameters for each unit cell.

Figure S2 and S3 show the strain in y-direction for different applied global displacements ($u_{x,1} < u_{x,2} < u_{x,3}$ ...). Figure S2 is a result of the homogenized examples with variations in the parameter α (left) and μ_g (right). The established final shape is marked in dark-red. Figure S3 (right) shows the applied displacement and resulting reaction forces dependent on the simulated time step for the non-linear structures. The FEM simulations as well as the semi-analytical model have been used to characterize the structure of the modified unit cell (Figure S1b) with two different distributions of the parameter t_2. Figure S3 (top) shows the results for a linear distribution and Figure S3 (bottom) for a sinusoidal. The semi-analytical model roughly approaches the simulation results, but differs with increasing displacement. The end shapes (dark-red lines) do not differ, but we see that the intermediate shapes (the way the shape is changing during load) can be controlled with the parameter distribution.

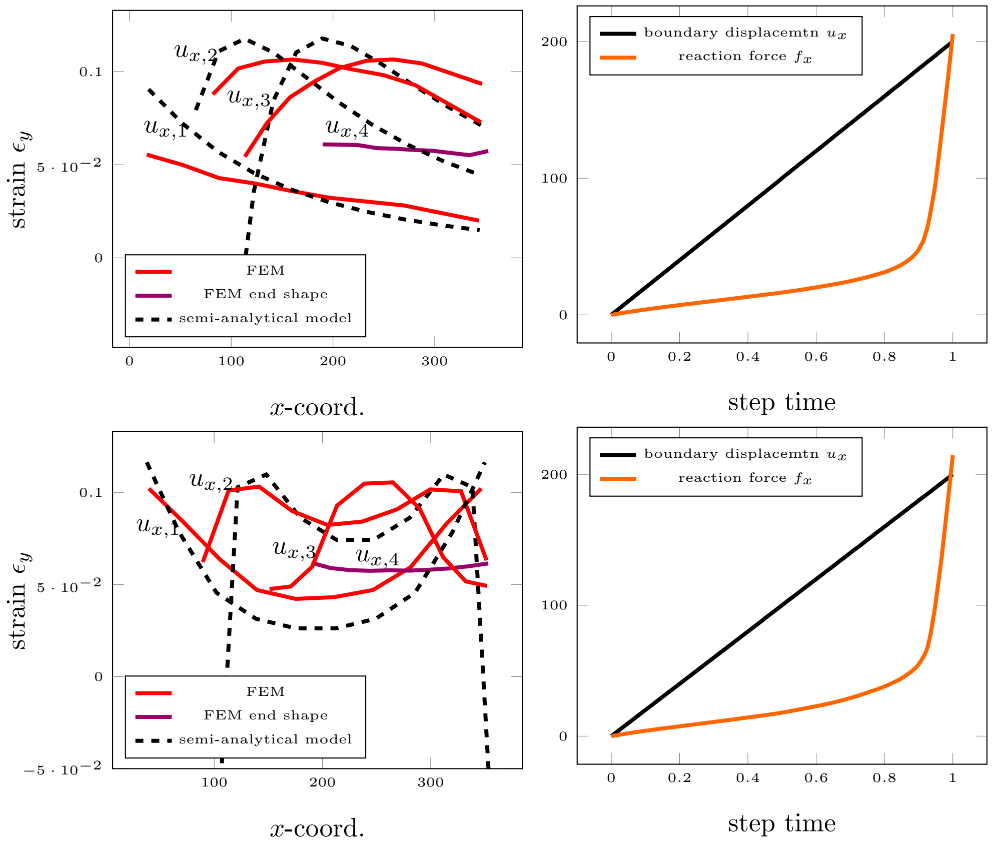

Figure S3: Lateral-strain dependent on the axial strain for two different hinge thickness distributions over the x-coordinate of the samples (left). The locally different stiffnesses lead to a varying lateral strain over the x-coord. of the sample. In addition this lateral strain is non-linearly dependent on the axial strain. Different hinge distributions leads to different global material behavior (linear distribution shown on top and sinusoidal distribution on bottom). The results have been calculated semi-analytically (dotted lines) and validated with FEM simulations (red lines). The right graph shows the applied displacement and resulting forces over the simulation time step.

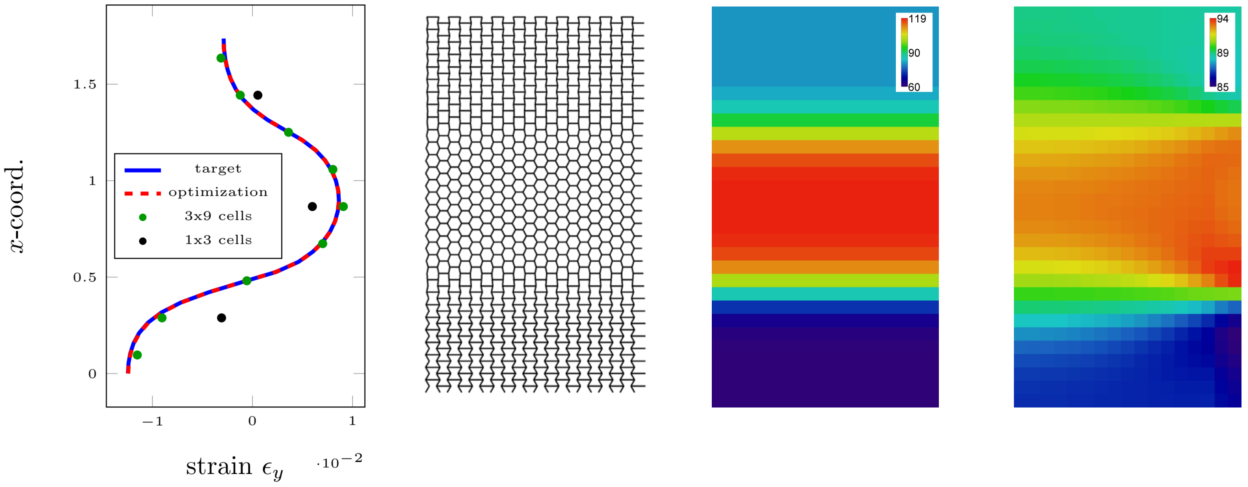

Figure S4: Comparison of target and simulated lateral strain (left), schematic micro structure (middle left) and distributions of the angle α in the material: original (middle right) and optimized (right) α.

S3. Optimization

In Figure 3c in the main part we showed results of optimizing geometrical parameters in unit cells in a macroscopic material. Here, we provide further information on the optimization process and validate the results. Note that the optimization is constrained by equations of mechanical equilibrium or, equivalently, the mechanical boundary value problem. In order to compute improved parameter values γ, we have to compute directional derivatives or sensitivities. Applying the standard sensitivity approach, we have to solve for each unit cell in the compound a non-linear system of equations. After linearization and using finite differences, one must solve the same amount of equations plus one, but now they are of linear type. A better alternative here is the adjoint sensitivity approach: Before each gradient descent iteration the original mechanical balance of equilibrium and its adjoint version have to be solved, in total two systems of equations. Still, the differentiation w.r.t. γ is inevitable. For this problem, however, we may use numerical or analytical homogenization and a subsequent interpolation of sufficiently high order between different parameter values. This approach is also applicable to a optimization problem with the unit cell in Figure 3 (main section), since an analytical description of the material response (for unit cells in array compound) is hardly possible.
To validate our optimization models for the angle α, we compared the target displacement, results of the homogeneous model and a FEM beam model of the whole structure. First we compared structures with different numbers of unit cells to the homogeneous model. We found good accordance when the complete structure consist out of at least 3 x 9 unit cells (see Figure S4 (left)). Also, the given target strain could be reached with the optimized parameter distribution with fault tolerance of 10^{-4}. The parameter range could be reduced from 60° to 10° compared to the manually chosen distribution (see Figure S4 (right)). We also optimized the structure with contact gaps and to find the optimal distribution of the gap width μ_g. In Figure S5 (left) we compared the optimized model to the target function and validated our model. The fault tolerance was $8 \cdot 10^{-6}$. Parameter distributions for chosen-by-hand and optimized model are shown in Figure S5 (right) but do not differ that much. Figure S4 and S5 (middle left) show the resulting microstructures schematically

S4. Manufacturing

The demonstrator part (Figure S6) was produced with the additive manufacturing process *ARBURG Plastic Freeforming (APF)* using the thermoplastic elastomer (TPE) *Elastollan C78 A15* and a nozzle with diameter 0.2 mm. The dimensions were $x = 185.7$ mm, $y = 44.8$ mm, $z = 16$ mm. The slicing was done with a layer hight of 0.2 mm, a discharge number of 60% and a droplet form factor of 1.295. The printing speed was 65 $mm\ s^{-1}$ for continuous extrusion and 20 $mm\ s^{-1}$ for discrete extrusion. The infill density was 100% and 1 perimeter was used. The infill was done with an overlap of 50% to the perimeter with a starting angle of 45° and an increment angle of 90°. The temperatures were 35°C at the ma-

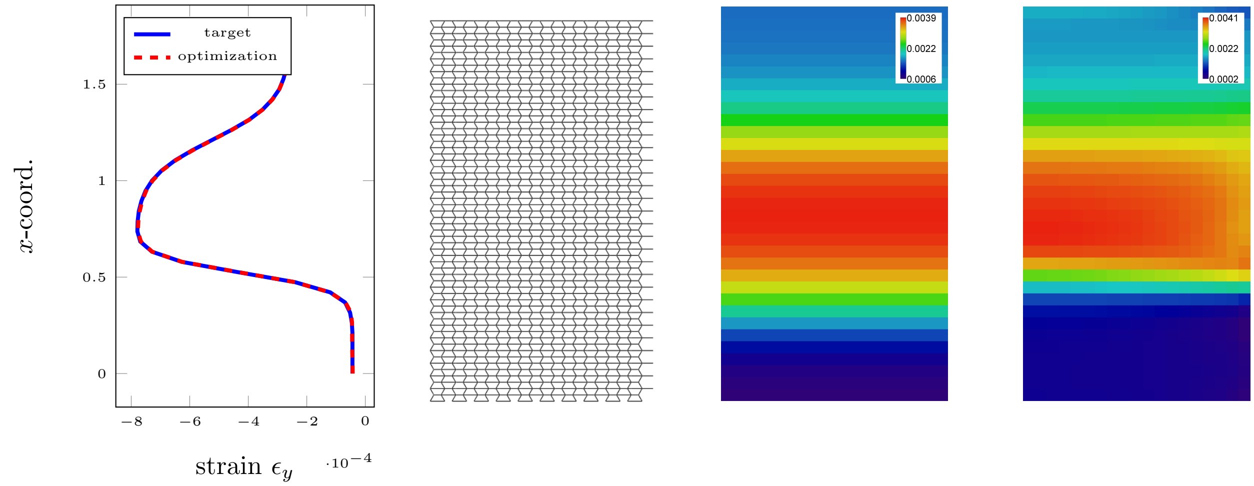

Figure S5: Comparison of target and simulated lateral strain (left), schematic micro structure (middle left) and distributions of the contact gapμ_g within the material: original (middle right) and optimized (right) μ_g.

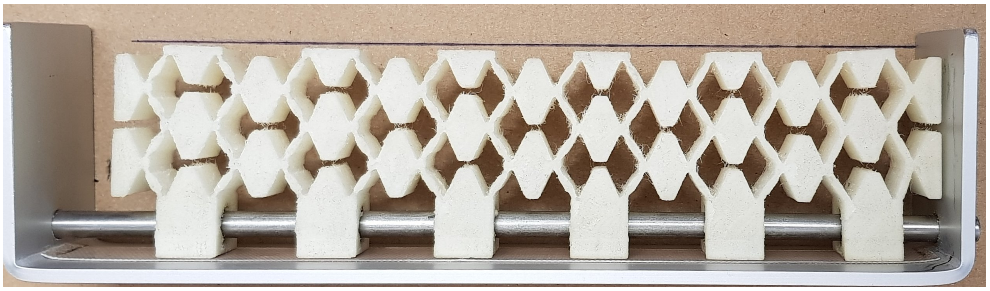

Figure S6: 3D-printed sample.

terial feeding zone, 150°C at zone one, 170°C at zone two, 215°C at the discharge head and an building chamber temperature of 90°C. To analyze the behavior, pictures were taken under different boundary displacements (see Figure 5b, main part). The obtained lateral deformations were evaluated optically and resulting local strains were calculated out of the shift of the pixels (see Figure 5c, main part and video S7).
